# Multidisciplinary evaluation of plant growth promoting rhizobacteria on soil microbiome and strawberry quality

**DOI:** 10.1186/s13568-023-01524-z

**Published:** 2023-02-16

**Authors:** Jun Haeng Nam, Alyssa Thibodeau, Yanping L. Qian, Michael C. Qian, Si Hong Park

**Affiliations:** Department of Food Science and Technology, Oregon State University, 3051 SW Campus Way, Corvallis, OR 97331 USA

**Keywords:** Plant growth promoting rhizobacteria, Strawberry, Soil microbiome, Biofertilizer

## Abstract

The natural soil environment is considered one of the most diverse habitats containing numerous bacteria, fungi, and larger organisms such as nematodes, insects, or rodents. Rhizosphere bacteria play vital roles in plant nutrition and the growth promotion of their host plant. The aim of this study was to evaluate the effects of three plant growth-promoting rhizobacteria (PGPR), *Bacillus subtilis*, *Bacillus amyloliquefaciens*, and *Pseudomonas monteilii* for their potential role as a biofertilizer. The effect of the PGPR was examined at a commercial strawberry farm in Dayton, Oregon. The PGPR were applied to the soil of the strawberry (*Fragaria* × *ananassa* cultivar Hood) plants in two different concentrations of PGPR, T1 (0.24% PGPR) and T2 (0.48% PGPR), and C (no PGPR). A total of 450 samples from August 2020 to May 2021 were collected, and microbiome sequencing based on the V4 region of the 16S rRNA gene was conducted. The strawberry quality was measured by sensory evaluation, total acidity (TA), total soluble solids (TSS), color (lightness and chroma), and volatile compounds. Application of the PGPR significantly increased the populations of *Bacillus* and *Pseudomonas* and promoted the growth of nitrogen-fixing bacteria. The TSS and color evaluation showed that the PGPR presumptively behaved as a ripening enhancer. The PGPR contributed to the production of fruit-related volatile compounds, while the sensory evaluation did not show significant differences among the three groups. The major finding of this study suggests that the consortium of the three PGPR have a potential role as a biofertilizer by supporting the growth of other microorganisms (nitrogen-fixing bacteria) as part of a synergetic effect and strawberry quality such as sweetness and volatile compounds.

**Supplementary Information:**

The online version contains supplementary material available at 10.1186/s13568-023-01524-z.

## Key points

- Application of the PGPR significantly increased the population of the *Bacillus* and *Pseudomonas* genus.
- Application of the PGPR induced the growth of other beneficial bacteria.
- The PGPR have potential role as a ripening enhancer.

**Supplementary Information:**

The online version contains supplementary material available at 10.1186/s13568-023-01524-z.

## Introduction

A complex biological and chemical activity of microbes in the soil contributes to the quality and productivity of fresh strawberries (Jung et al. 2012; Jacoby et al. 2017; Lazcano et al. 2021; Liu et al. 2021). These factors not only help develop natural plant defenses, but help to resist stresses caused by biotic and abiotic factors (Jung et al. 2012). Organic produce farming is a type of farming that avoids or limits the use of synthetic fertilizers, growth regulators, and livestock feed additives. Organic farmers instead rely heavily on biofertilizers, crop rotations, compost, cover crops, plant by-products, animal manure, and other biologic materials to enhance product quality (Samtani et al. 2019).

The use of harmful chemical fertilizers and pesticides is an ongoing issue that has significant negative impacts on the environment (Pahalvi et al. 2021). These threats have induced interest in using beneficial microbes, such as PGPR (plant growth-promoting rhizobacteria), to develop sustainable and safe agricultural practices (Alori and Babalola 2018; Deng et al. 2019; Liu et al. 2021). Biofertilizers selectively maintain soil microbiota that may be employed as microbial or soil inoculants to increase both plant and soil fertility and plant productivity (Pereg and McMillan 2015; Deng et al. 2019; Liu et al. 2021). In other words, a biofertilizer or microbial fertilizer, is a substance composed of living microbes and a combination of biodegradable substances that enhance the growth and yield by increasing availability of essential nutrients to the host plant. The biofertilizers applied to seed, plant surfaces, or soil, colonize the plant’s environment through several means, such as rhizosphere and intercellular spaces (Pereg and McMillan 2015; Deng et al. 2019).

The PGPR are microbes associated with the plant rhizosphere (the space around the plant roots) where coordinate with the plant to exchange nutrients (Deng et al. 2019; Lyu et al. 2019; Vejan et al. 2016). Many bacterial species, most of which are present in the plant rhizosphere, have been examined and suggested to be beneficial to plant growth, yield, and crop quality (Lazcano et al. 2021). In general, PGPR includes strains in the genera *Pseudomonas*, *Bacillus*, *Azotobacter*, *Erwinia*, *Serratia*, *Azospirillum*, *Caulobacter*, *Chromobacterium*, *Agrobacterium*, *Flavobacterium*, *Arthrobacter*, *Micrococcus*, and *Burkholderia* (Miransari 2016; Vejan et al. 2016; Di Benedetto., 2017; Verma et al. 2019). Specifically, *Bacillus* species produce spores that can live in the soil for lengthy periods in adverse environments (Hashem et al. 2019). Though the exact processes through which PGPR stimulates plant growth are not fully understood, both direct and indirect mechanisms are suggested. A review published by Hashem et al. (2019) suggests that PGPR stimulate plant growth by inducing systemic resistance against biotic stress, antibiosis, and competitive omission.

Several approaches such as culture-dependent and -independent methods have been established to examine microbes in the soil and rhizosphere (Lee et al. 2021; Rincon-Florez et al. 2013; Romano et al. 2020; Sangiorgio et al. 2022). Though the culture-dependent methods are widely applied to study the persistence of microbes, it is challenging to distinguish overall microbes based on morphological characteristics. Moreover, there are still many unculturable microbes (Romano et al. 2020). Next generation sequencing (NGS) techniques are culture-independent methods that may provide a more direct identification of microbial taxa and overcome the limitations of culture-dependent methods (Oberauner et al. 2013; Uroz et al. 2013; Zhu et al. 2018). Considering the plant rhizosphere includes a complex assembly of diverse microbes and the potential impact of indigenous microbes, investigating the soil microbiome can provide a deeper understanding of PGPR effects (Deng et al. 2019; Xiong et al. 2021). Microbiome sequencing can identify the transmission of indicator microbes from environmental sources in the soil as well as the shift of microbial populations due to PGPR treated rhizospheres.

In this study, a consortium of three PGPR: *Bacillus subtilis*, *Bacillus amyloliquefaciens,* and *Pseudomonas monteilii* was evaluated for its potential roles as a biofertilizer through microbiome and strawberry quality analysis. The PGPR were applied to the soil of strawberry (*Fragaria* × *ananassa* cultivar Hood) plants to profile the main soil rhizobacteria and microbial diversity associated with the PGPR treatment. Additionally, sensory evaluation, pomological (total acidity (TA), total soluble solid content (TSS), and color (lightness and chroma)) assays, and volatile compounds of the strawberries harvested from PGPR treated soil were measured to evaluate strawberry quality.

## Materials and methods

### Experimental design and sample collection

**Fig. 1:**
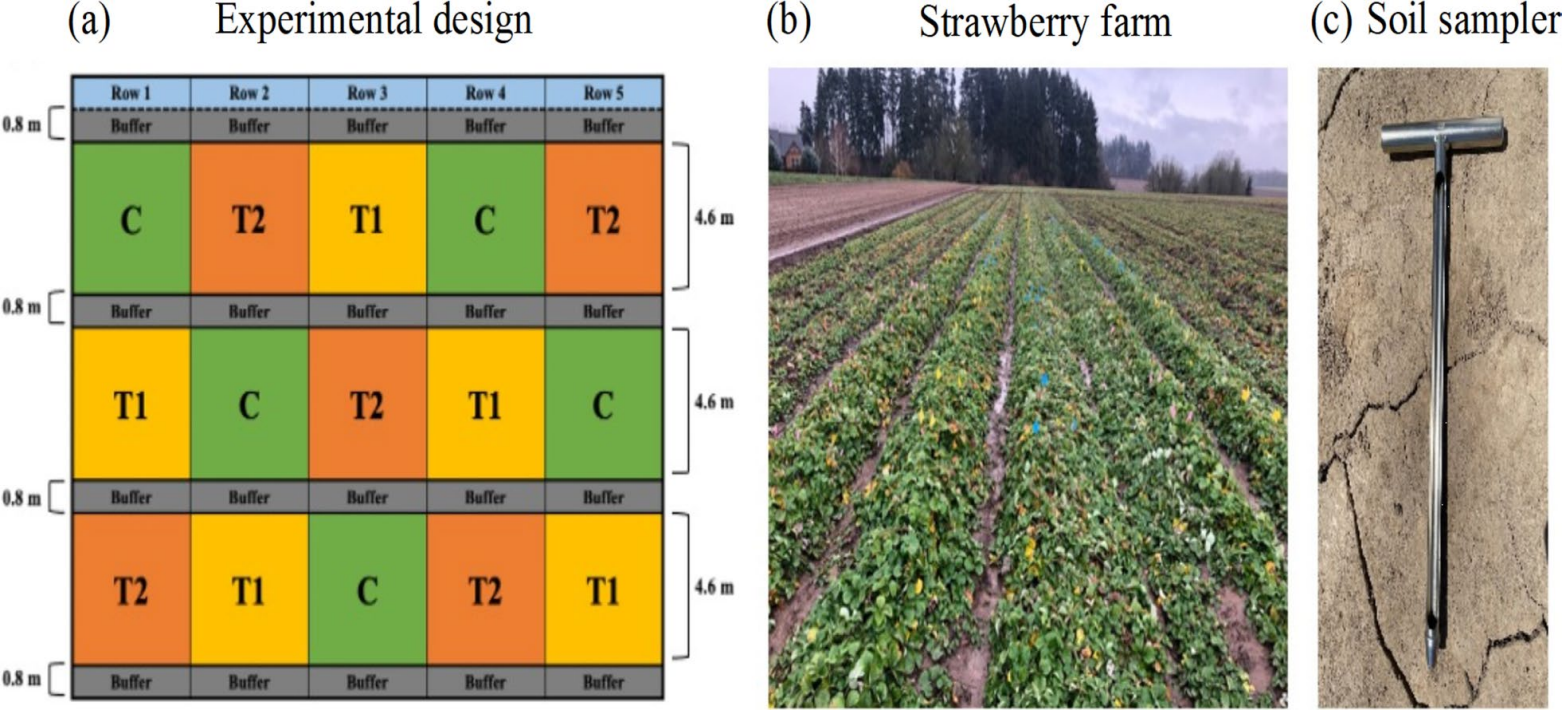
Experimental design for this study. **a** Schematic of the experimental design, **b** The experimental site located in Dayton, OR, **c** 21′′ long sampler probe for soil sample collection. C (no PGPR); T1 (0.24% PGPR); T2 (0.48% PGPR). Different letters indicate significant difference (*p* < 0.05)

The Soil Activator^™^ (Earth Alive^™^, Lasalle, QC, Canada), containing three PGPR species: *B. subtilis*, *B. amyloliquefaciens*, and *P. monteilii*, was used in this study. The experimental field was located on a commercial strawberry farm in Dayton, Oregon (45.23475°N, 123.04942°E, USA) and included a total of 15 plots (five rows (1 × 16.2 m) with each row having three plots (1 × 4.6 m per plot)). Buffered zones (1 × 0.8 m) were located at the end of each row and between the plots to minimize overlap between the plots. The 15 plots were divided into 3 different groups with different PGPR concentrations: Control (C, 0% PGPR); Treatment 1 (T1, 0.24% PGPR); Treatment 2 (T2, 0.48% PGPR) (Fig. 1a). The PGPR solution was applied bi-weekly to the soil of each designated plot during the experimental period (August 2020 to May 2021) and soil samples were collected monthly (10 sampling periods) (Fig. 1b). A total of 450 soil samples around the plant rhizospheres were collected [45 samples per month (15 samples per group)] throughout the sampling period using a 0.5 m long soil sampling probe (Fig. 1c). Soil samples were transferred to sterile 50 ml tubes, kept in an ice cooler at the time of sampling, and stored at − 80 °C until DNA extraction.

### Confirmation of PGPR

To confirm the presence of the three PGPR species: *B. subtilis*, *B. amyloliquefaciens*, and *P. monteilii* in the Soil Activator™ used in the study, a PCR assay and Sanger sequencing were applied using three PGPR specific primer pairs. Two primer pairs for *B. subtilis* and *B. amyloliquefaciens* were adopted from previous reports and a primer pair for *P. monteilii* was designed in this study (Additional file 1: Table S1). The Soil Activator^™^ was serially diluted with DNase-RNase free water, spreaded on Luria broth (LB) agar (Hardy Diagnostics, Santa Maria, CA, USA) for *B. subtilis* and *B. amyloliquefaciens* and MacConkey agar (Neogen, Lansing, MI, USA) for *P. monteilii*, respectively, and incubated at 37 °C for 24 h. The colonies were resuspended in 100 µL of DNase-RNase free water conducted a colony PCR assay. The PCR was performed in 25 µL total volume, containing 1 µL of the homogenized colony solution, 12.5 µL of Takara Premix Ex *Taq*^™^ (Takara^™^, Fisher Scientific, Pittsburgh, PA, USA), 400 nM of each forward and reverse primer, and 8.5 µL of DNase-RNase free water. The PCR assay conditions were varied and listed in the Additional file 1: Table S2. The PCR products were run on 3% agarose gels stained with Gel Red Dye (10 mg/mL) (Biotium, Fremont, CA, USA) and visualized using a Molecular Imager^™^ Gel Doc XR+© Imaging System (Cambridge Scientific Products, Waterland, MA, USA).

Each PCR product for *B. subtilis*, *B. amyloliquefaciens*, or *P. monteilii* was purified using a QIAquick PCR Purification Kit (Qiagen, Hilden, Germany) following the provided manual. The purified PCR products were sequenced at the Center for Quantitative Life Sciences (CQLS) at Oregon State University (OSU) (Corvallis, OR, USA) and confirmed using Basic Local Alignment Search Tool (BLAST) from National Center for Biotechnology Information (NCBI).

### DNA extraction from soil

Bacterial DNA was extracted from 0.25 g of the soil using a DNeasy PowerSoil Pro Kit (Qiagen) according to the protocol. The concentration of the isolated DNA was measured with a Qubit 4 Fluorometer (Thermo Fisher Scientific, Waltham, MA, USA) and each of the samples was diluted to a final concentration of 10 ng/µL using DNase-RNase free water.

### 16S rRNA library preparation

The sequencing library was generated targeting the V4 region of the 16S rRNA gene according to the previous report (Kozich et al., 2013). Briefly, the extracted DNA from each sample was amplified using a high-fidelity AccuPrime^TM^ *pfx* Super Mix (Thermo Fisher Scientific). Amplified DNA fragments were confirmed through 1% agarose gel electrophoresis and normalized using a SequalPrep^™^ Normalization Kit (Thermo Fisher Scientific) following the manufacturer’s recommendation. A library pool for sequencing was generated by combining 5 µL of each normalized sample and concentration was quantified with a Qubit 4 Fluorometer (Thermo Fisher Scientific). The pooled library was loaded on the cartridge v2. (2 × 250 bp, 500 cycles) and sequenced using an Illumina MiSeq at the CQLS at OSU (Corvallis).

### Microbiome data analysis

The raw sequences were downloaded from the Illumina BaseSpace website (https://basespace.illumina.com) and further processed with The Quantitative Insights into Microbial Ecology 2 (QIIME 2) version 2021. 04 (Bolyen et al. 2019). The raw sequences were demultiplexed and quality filtered using the q2-demux plugin, followed by DADA2 (Callahan et al. 2016). An operational taxonomic unit (OTU) table was then generated and the taxonomy of the sequenced data was obtained using Silva 132 reference database (Quast et al. 2013; Yilmaz et al. 2014; Glöckner et al. 2017). The obtained taxonomic table was further processed with MicrobiomeAnalyst (http://www.microbiomeanalyst.ca) to generate alpha and beta diversity plots (Dhariwal et al. 2017; Chong et al. 2020). Analysis of variance (ANOVA) and analysis of similarity (ANOSIM) tests were applied to evaluate the significant differences for alpha and beta diversity, respectively. The taxonomic data were also applied to a heatmap generation to explore the relative abundance of bacteria among samples (Hunter 2007).

### Strawberry harvesting

The strawberries were harvested on the morning of June 3, 2021. Approximately 34 kg of strawberries were harvested from the entire experimental plot (11.3 kg from each treatment group; C, T1, and T2). The harvested strawberries were distributed into disposable plastic containers based on cultivated plot and treatment group. The harvested strawberries were kept in a cooler and directly delivered to the OSU Center for Sensory and Consumer Behavior Research for the sensory evaluation that was performed on the same day.

### Pomological analysis

To perform the TA and TSS (Brix) evaluation, 30 of the harvested whole strawberries from each group were distributed to three sub-groups (10 strawberries per sub-group) and homogenized using an Oster^®^ Precise Blender 200 (Oster^®^, Boca Raton, Florida, USA). The TSS was measured in triplicates with a digital refractometer, RFM 81 (Bellingham and Stanley, Tunbridge Wells, UK) with the homogenized strawberries (AOAC 1998).

The TA was measured in triplicates by titrating 2 g of the homogenized strawberries with 0.1 N NaOH (pH 8.2) using an automatic titrator (Orion Start T910, Thermo Fisher Scientific) (AOAC 1998). The amount of titrant needed to reach pH 8.1 was noted and the titratable acidity (% citric acid) was calculated according to the following equation:

$$\frac{{{\text{Volume of NaOH}} \times {\text{Normality of NaOH}} \times {\text{Acid factor}} \times 100}}{{\text{Weight of the sample}}}$$

The color was measured at the center of the flat surface of the 30 harvested strawberries, using a Hunter Labscan spectrophotometer (MS/S-4500L, Hunter Associates Laboratory Inc., Reston, VA, USA) (Xie and Zhao 2004). Color values: L* (lightness); a* [green ( − ) to red (+)]; b* [blue ( − ) to yellow (+)] were measured. Both a* and b* values were used to calculate hue angle [arctan (b*/a*)] and chroma [0.5 (a*2+b*2)], which were compared to determine color variance among the strawberries from each treatment group.

### Volatile compounds analysis

Approximately 30 g of the harvested strawberries from each treatment group was homogenized into a paste. Two grams of the paste was weighed into a 20 mL vial and mixed with 8 ml of citrate buffer, which was formulated with 0.2 M, pH 3.2, 1% NaF (saturated with NaCl), and 20 µL of 4-octanol as an internal standard (IS) was added. The gas chromatography/mass spectrophotometry (GC/MS) was used to identify the volatile compounds. For the assay, an Agilent 7890 GC attached with an Agilent 5975 mass selective detector (MSD, Agilent Technologies, Santa Clara, CA, USA) and a Gerstel MPS2 (Gerstel Inc. Linthicum, MD, USA) was used. The sample was equilibrated at 45 °C for 2 min. After equilibration, headspace volatiles were collected on a 2 cm three-phase solid phase microextraction (SPME) fiber coated with divinylbenzene/carboxen/polydimethylsiloxane (DVB/ CAR/PDMS, 50/30 μm film thickness, Supelco, Bellefonte, PA, USA) for 40 min at 45 °C. After extraction, volatile desorption was performed by introducing the SPME fiber into a GC injection port at 250 °C in a spitless mode for 5 min. An autosampler controlled all the steps (Gestel, INC., Linthicum, MD, USA).

For chromatographic separation, a ZB-Wax column (60 m length, 0.25 mm i.d., 0.5 μm film thickness Phenomenex, Inc., Torrance, CA, USA) was used. The column flow rate (helium) was 1.5 mL/min. The initial oven temperature was 40 °C and held for 4 min, then ramped to 230 °C at a rate of 4 °C/min, with a 10 min holding. Injection port, MS transfer line, and ion source temperatures were 250, 280, and 230 °C, respectively. Electron ionization mass spectrometric data from m/z 35 to 350 were collected, with an ionization voltage of 70 eV. Compound identifications were made by comparing mass spectral data samples with the Wiley 275 L database (Agilent Technology, Qian et al. 2019). The results were further analyzed with an Enhanced Chemstation software E.02 (Agilent Technologies).

### Sensory evaluation

The objective of the sensory test was to determine consumer acceptance of the harvested strawberries from the C, T1, and T2 groups. A total of 52 participants (24 females and 28 males, ages 18 to 65 years) were recruited from the community based on an online survey. The participants were regular strawberry consumers and had no known food allergies. Five strawberries per group were placed on 6-inch white paper plates labeled with 3-digit blinding codes. Panelists were asked a series of liking/disliking questions (overall liking, appearance liking, flavor liking, and texture liking) using a 9-point hedonic scale (1=dislike extremely, 9=like extremely) as well as a series of intensity-related questions (sweetness, tartness, and firmness) using a 5-point just-about-right (JAR) scale (1=not strong enough, 3=JAR, 5=much too strong). The test was performed in individual booths using computerized ballots (Compusense Cloud, Compusense Inc., Guelph, ON, Canada) at the sensory testing facility at Oregon State University. The study protocol was reviewed and approved by the Oregon State University’s Institutional Review Board (IRB) (IRB# 2021-0953). All subjects gave written informed consent and were paid for participation.

### Statistical analysis

The data obtained from the microbiome and pomological assays (TSS, TA, and color) were analyzed for statistical differences with Minitab 16 (Minitab, State College, PA, USA). All statistical analyses were performed using ANOVA to determine if there was a difference between treatment groups, followed by Tukey’s post hoc test to distinguish where specific differences were present. The significant difference between groups was determined based on the *p*-value < 0.05.

## Results

### Prevalence of the three PGPR

**Fig. 2:**
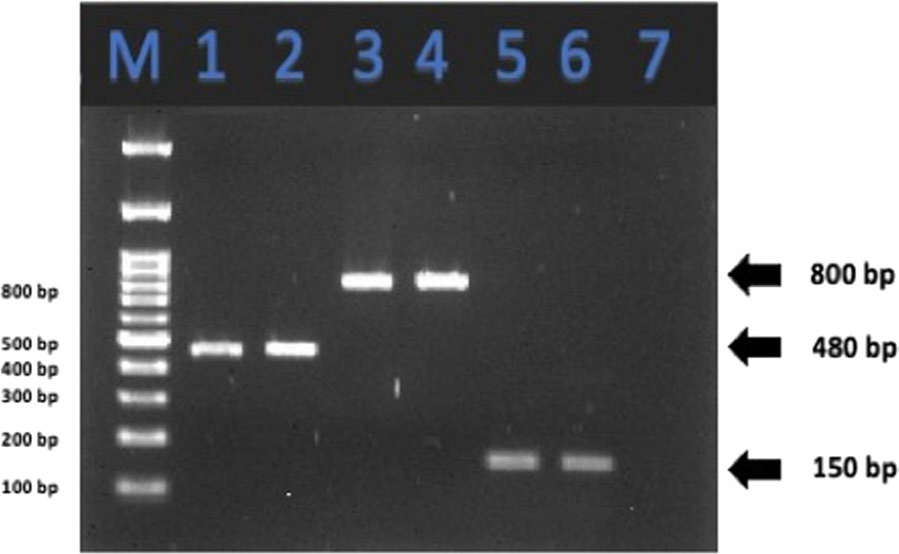
The PCR assay detecting the presence of *B. subtilis* (480 bp, lanes 1 and 2), *B. amyloliquefaciens* (800 bp, lanes 3 and 4), *P. monteilii* (150 bp, lanes 5 and 6). 100 bp DNA ladder (lane M), and negative control (lane 7)

The PCR assay was performed and confirmed the presence of the three PGPR in the commercial product (Soil Activator^™^, Fig. 2). To further confirm, the sequences obtained from the PCR products were compared using a NCBI BLAST. The sequences showed a high similarity with the target bacterium; 99.3, 100, and 90.6% for *B. subtilis*, *B. amyloliquefaciens*, and *P. monteilii*, respectively.

### Microbiome analysis

**Fig. 3:**
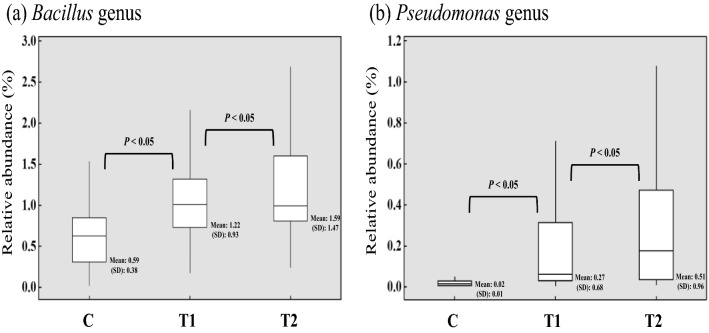
Box plot comparing relative abundance of the two PGPR genus (**a**) *Bacillus* (**b**) *Pseudomonas* in the soil samples collected from C (no PGPR); T1 (0.24% PGPR); T2 (0.48% PGPR). Different letters indicate significant difference (*p* < 0.05)

The microbiome analysis of soil samples collected from each treatment group during the sampling period was performed. A total of 31,764,666 sequencing reads generated from 450 samples were obtained after the quality filtering with DADA2 (Callahan et al. 2016). The mean value of the frequency of sequences per sample was 70,588 and a total of 856 OTUs were identified to the genus level. The relative abundance of the PGPR genus, *Bacillus* and *Pseudomonas*, in the three groups was also observed and significantly different among the treatment and control groups (T1, T2, and C) (Fig. 3).

**Fig. 4:**
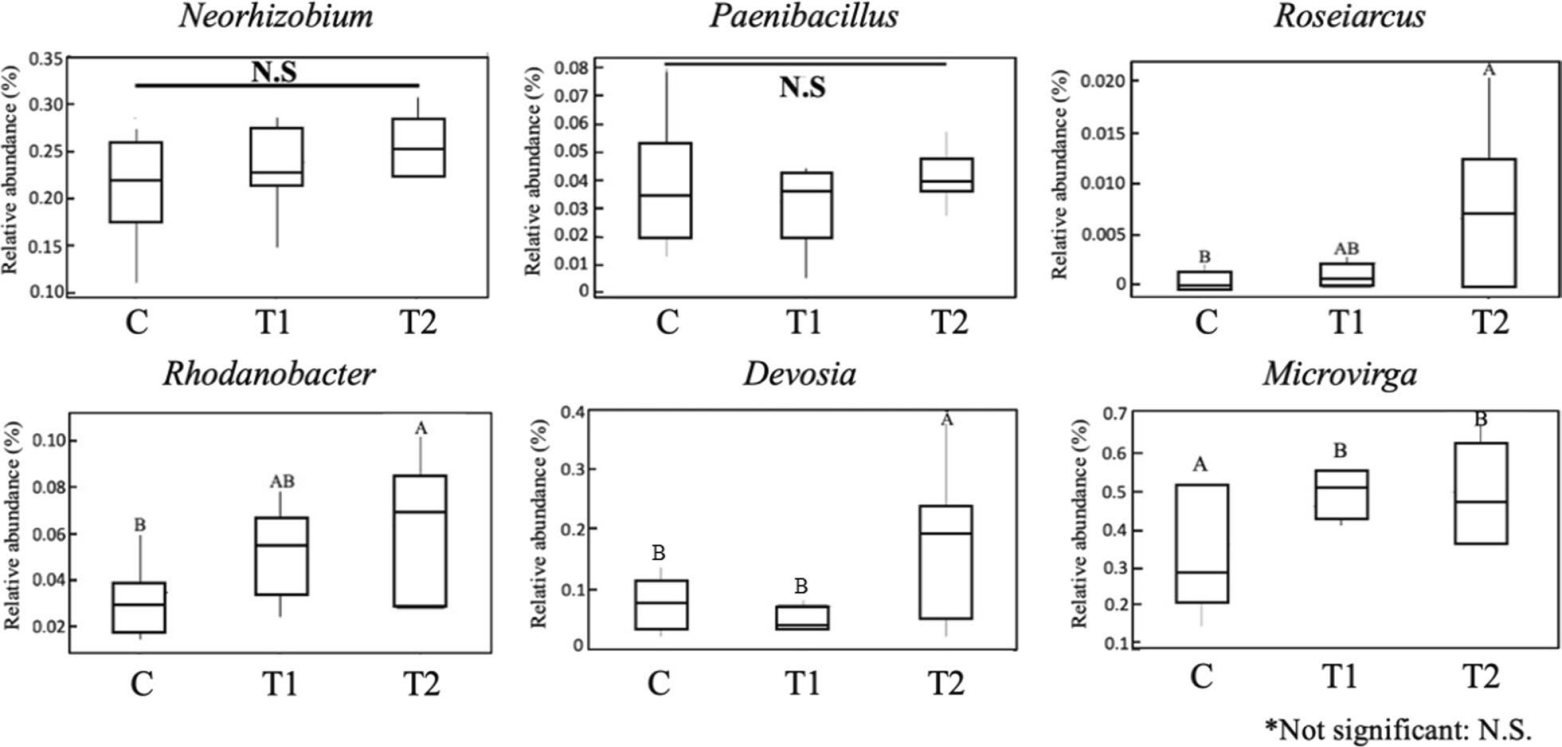
Relative abundance of the six nitrogen-fixing bacteria at genus level in the three treatment groups. C (no PGPR); T1 (0.24% PGPR); T2 (0.48% PGPR). Different letters indicate significant difference (*p* < 0.05)

Analysis of the taxonomic distribution, particular to the presence of nitrogen-fixing bacteria to the genus level (*Neorhizobium*, *Paenibacillus, Roseiarcus, Rhodanobacter, Devosia,* and *Microvirga*), was also conducted. The relative abundance of 4 nitrogen-fixing bacteria in the three different treatment groups was significantly different (*p* < 0.05) (Fig. 4). *Roseiarcus, Rhodanobacter,* and *Devosia* exhibited significantly higher abundance in the T2 group compared to the T1 and C groups, while *Microvirga* were significantly higher in the T1 and T2 groups compared to the C group (Fig. 4). However, the relative abundance of *Neorhizobium* and *Paenibacillus* among the three groups was not significantly different (Fig 4).

**Fig. 5:**
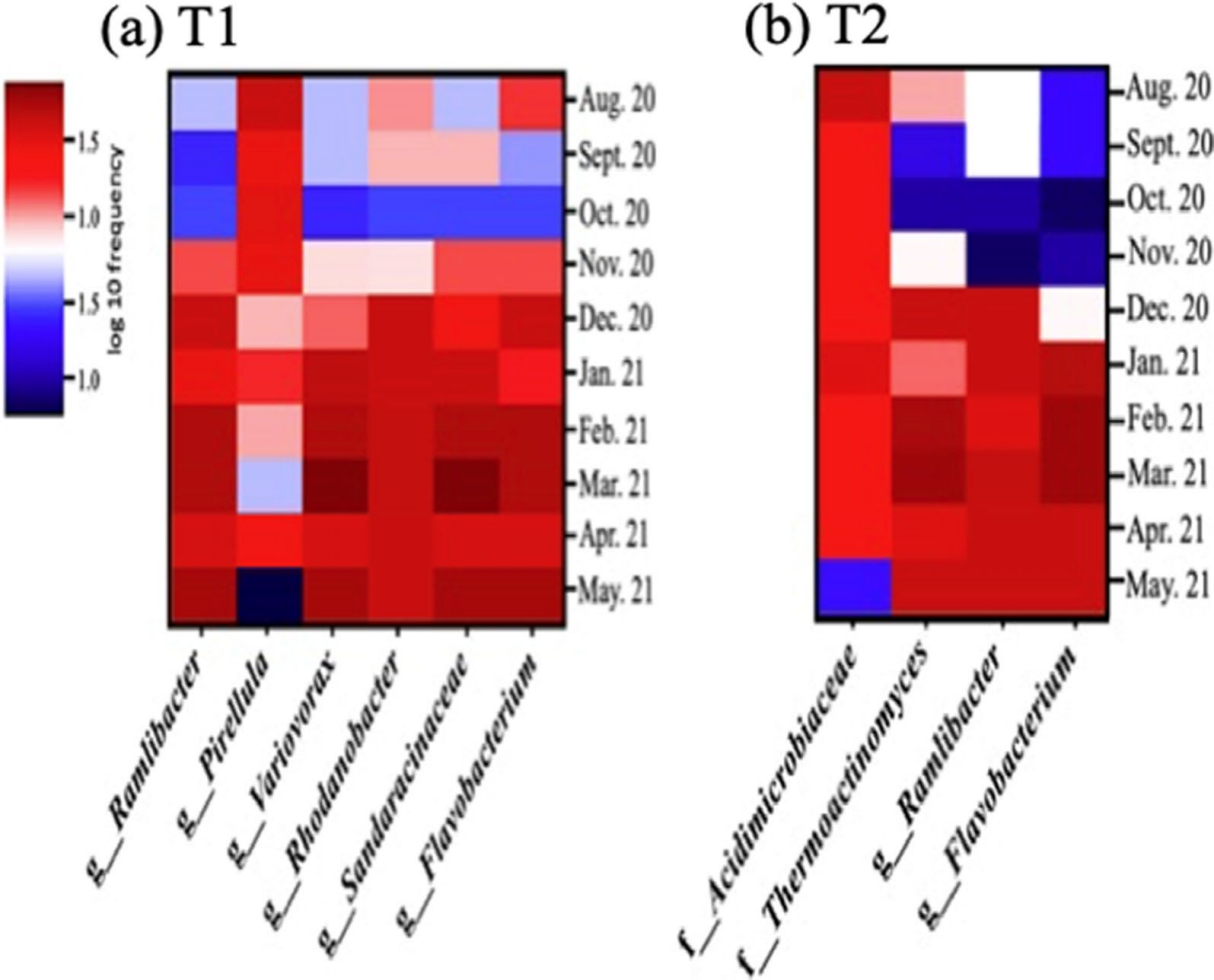
Heatmap expressing population shift of microorganism with soil related functions in the (**a**) T1 (0.24% PGPR) and (**b**) T2 (0.48% PGPR)

It is important to determine any soil microbial population shifts that could have had a positive impact on plant functions (i.e. improving tolerance against pathogens, degrading complex compounds, or reducing plant stress). To assess population shifts at the family or genus level, we created heat maps that spanned included the entire study period (Fig. 5). In the T1 group, the prevalence of *Ramlibacter, Variovorax, Rhodanobacter, Sandaracinaceae,* and *Flavobacterium* increased, while *Pirellula* was decreased (Fig 5a). In the T2 group, *Ramlibacter, Flavobacterium,* and *Thermoactinomyces* tended to increase over time generally, whereas the abundance of *Acidimicrobiaceae* was consistent, except the last month of sampling (May 2021) (Fig. 5b).

**Fig. 6:**
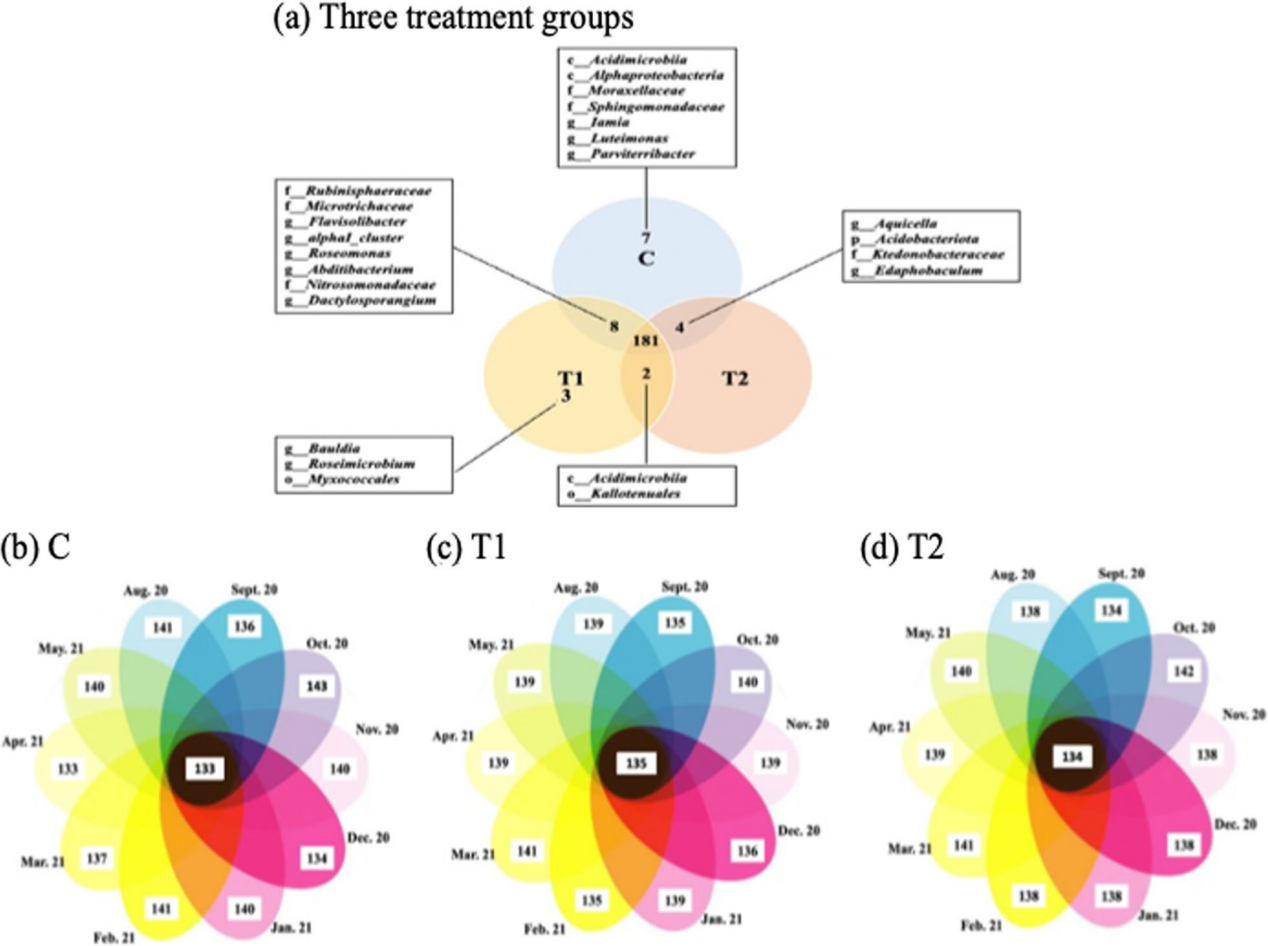
**a** A Venn diagram of shared and unique taxa among the three treatment groups. **b** Number of shared and unique taxa identified during the 10-month study period (August, 2020 to May, 2021) in C (no PGPR), **c** T1 (0.24% PGPR), and (**d**) T2 (0.48% PGPR)

The soil-associated core microbiome was identified because these taxa are the most likely to have influence on plant functions (Fig. 6). We defined the core microbiome based on spatial distribution (presence in two or more groups) and ecological stability (present at every sampling point). The total number of taxa shared by all groups was 181 (Fig. 6a). The taxa uniquely shared between only the C and T1 groups included *Rubinisphaeraceae*, *Microtrichaceae*, *Flavisolibacter*, *alphaI_cluster*, *Roseomonas*, *Abditibacterium*, *Nitrosomonadaceae*, and *Dactylosporangium*. The C and T2 groups uniquely shared *Aquicella*, *Acidobacteriota*, *Ktedonobacteraceae*, and *Edaphobaculum*, while the T1 and T2 group shared *Acidimicrobiia* and *Kallotenuales*. The identified unique taxa that appeared in the C and T1 group were *Acidimicrobiia*, *Alphaproteobacteria*, *Moraxellaceae*, *Sphingomonadaceae*, *Iamia*, *Luteimonas*, and *Parviterribacter* and *Bauldia*, *Roseimicrobium*, and *Myxococcales*, respectively (Fig. 6a). Shared taxa during the entire study period by month in the three groups were 133, 135, and 134 in C, T1, and T2 groups, respectively (Fig. 6b–d).

**Fig. 7:**
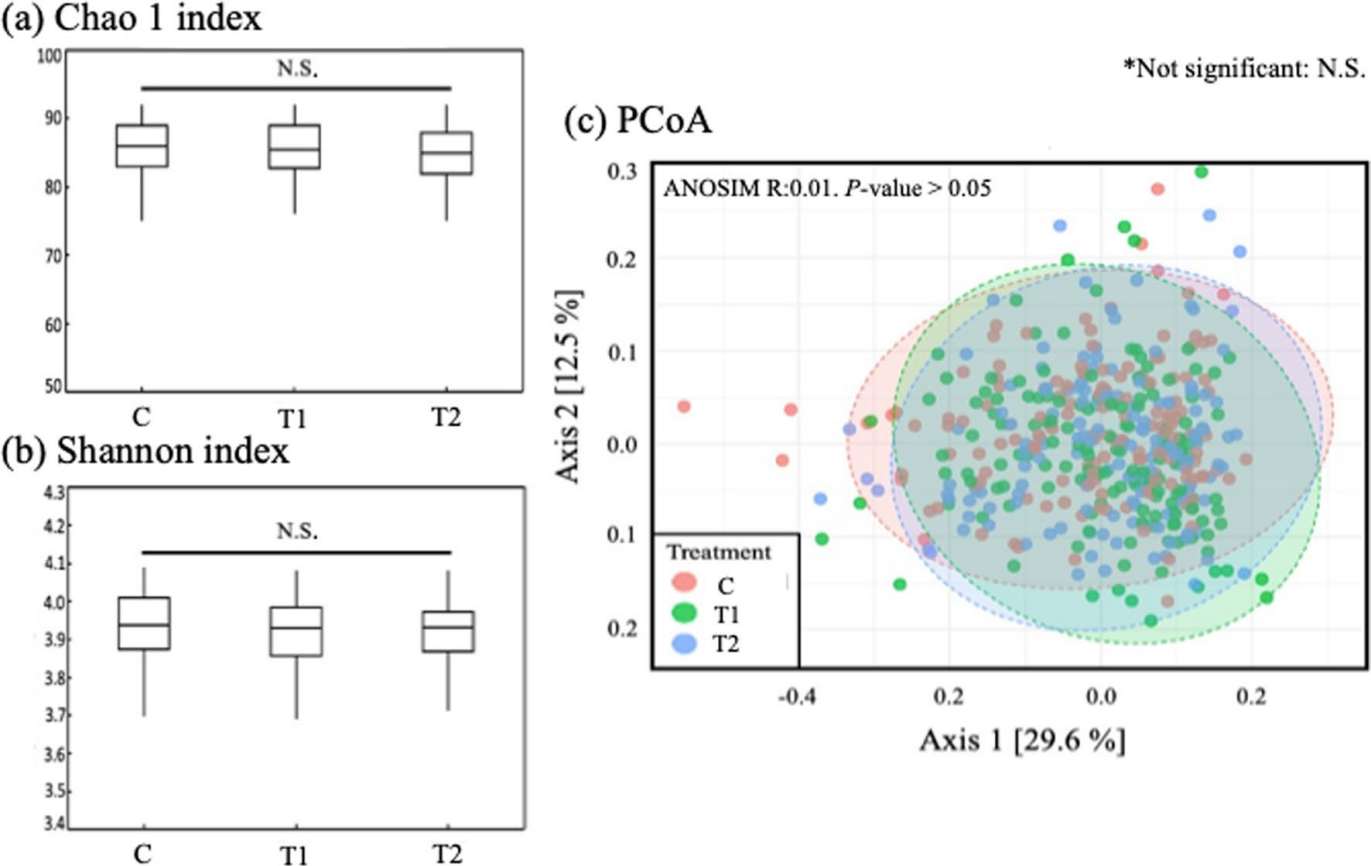
Alpha diversity. **a** Chao 1 index and **b** Shannon index of the three treatment groups. **c** Beta diversity and principal coordinates analysis (PCoA) originated from the Jaccard index model examining ANOSIM between the three treatment groups. C (no PGPR); T1 (0.24% PGPR); T2 (0.48% PGPR). Different letters indicate significant difference (*p* < 0.05)

Alpha diversity (species richness) in the different treatment groups was analyzed using Chao1 and Shannon indices (https://www.microbiomeanalyst.ca). Both indices did not indicate a significant difference among the three groups (*p* > 0.05) (Fig. 7a, b). Beta diversity was measured to assess variation in community composition among the samples and groups. The Jaccard index model was applied to examine Analysis of Similarities (ANOSIM) among the three different groups. The R-values obtained from ANOSIM vary between 0 and 1, and a lower R-value indicates higher similarity between groups. The *p*-value indicated there was no significant difference (*p* > 0.05) between the groups. The R-value (0.01) showed a high level of similarity between the groups (Fig. 7c).

### Pomological evaluation

**Fig. 8:**
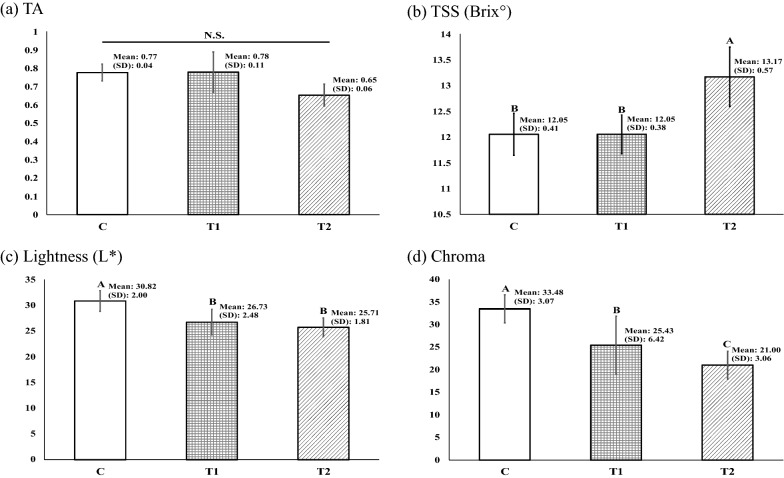
Pomological evaluation. **a** TA; **b** TSS; **c** lightness; **d** chroma of the strawberries cultivated from the three treatment groups. C (no PGPR); T1 (0.24% PGPR); T2 (0.48% PGPR). Different letters indicate significant difference (*p* < 0.05)

We used a pomological evaluation to investigate any effect of PGPR on strawberry quality. The TA, TSS, and color of the strawberries from each treatment group were evaluated. There was no significant difference of TA in the strawberries among the three groups (*p* > 0.05) (Fig. 8a). The strawberries from the T2 group were significantly higher in TSS compared to the C and T1 groups (*p* < 0.05) (Fig. 8b). For lightness evaluation, the strawberries from the T1 and T2 groups were significantly darker compared to the C group (*p* < 0.05) (Fig. 8c). The chroma value, which defines color purity, was significantly lower in the strawberries from the T1 and T2, compared to the C group (*p* < 0.05) (Fig. 8d).

### Volatile compound analysis

**Fig. 9:**
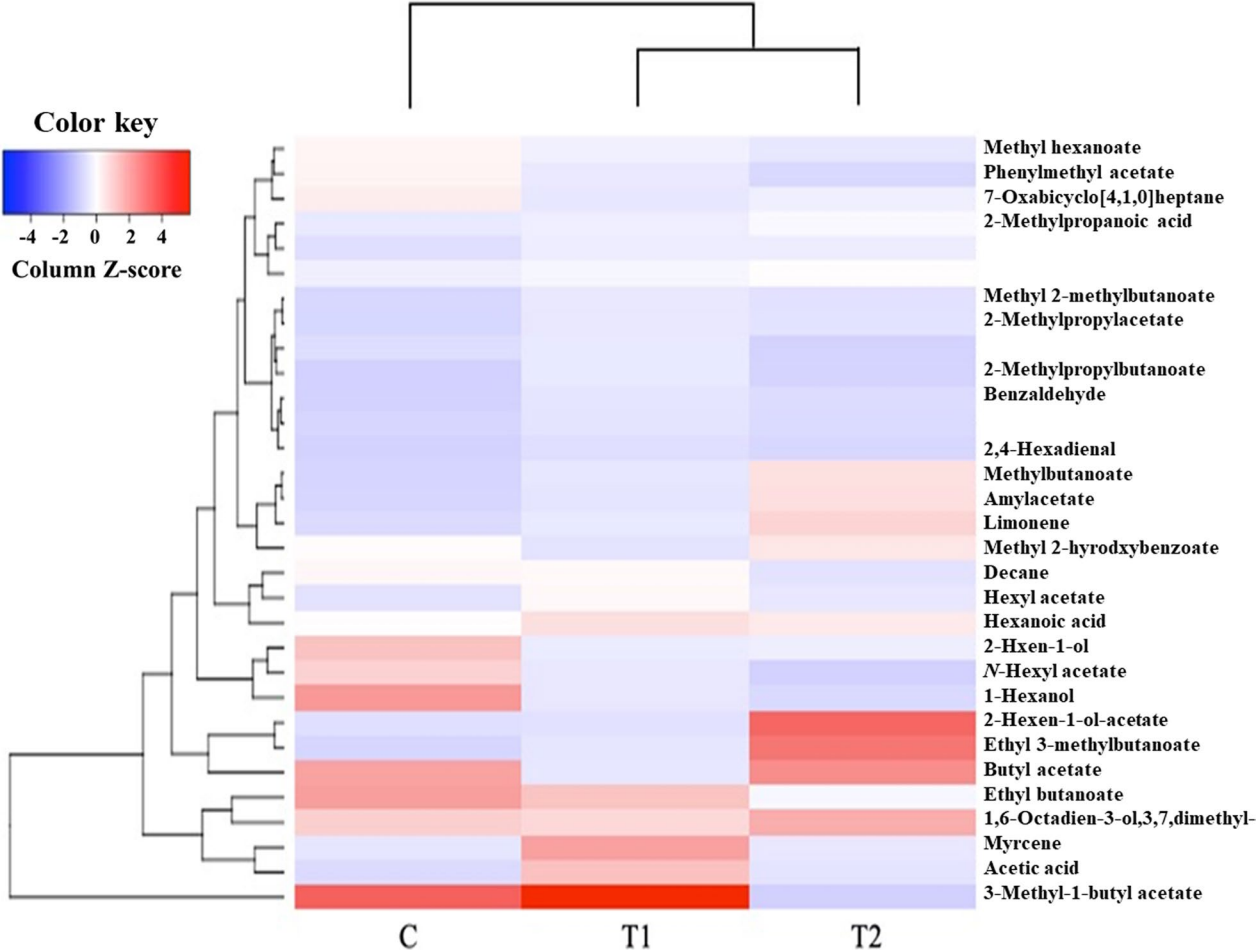
Heatmaps of PAR value of volatile compounds identified in the strawberries cultivated from the three treatment groups. Square box indicates the volatile compounds which are significantly higher in the T2 (*p* < 0.05). C (no PGPR); T1 (0.24% PGPR); T2 (0.48% PGPR)

Volatile compound contents in the strawberries grown with PGPR treated soil were evaluated using a GC/MS. A heatmap was generated with the peak area ratio (PAR) values of the volatile compounds shared in all three groups (Fig. 9). The volatile compounds related to fruit flavor (e.g., fruity/sweet, fresh, citrusy, and burnt sugar) were also investigated. Among the volatile compounds, the PAR values of methyl butanoate (fruity/sweet) and ethyl 3-methylbutanoate ester (fruity) were significantly higher in the strawberries from the T2 group (*p* < 0.05). Additionally, the PAR value butyl acetate (fruity) was significantly higher in both the T2 and C groups, when compared to T1. The PAR value of the other fruit-related volatile compounds, such as hexanal (green), and methyl hexanoate (fruity), were significantly higher in the strawberries from the C group (*p* < 0.05). The dendrogram indicated a closer relationship between the T1 and T2 groups compared to the C group.

### Sensory evaluation

The sensory evaluation of the strawberries cultivated from the three treatment groups was conducted with Liking and JAR evaluation. There were no significant differences in the overall appearance, flavor, and texture liking scores among the strawberries from the three groups (*p* > 0.05) (Table 1). Moreover, the result of JAR evaluation on sweetness, firmness, and tartness did not indicate a significant difference among the strawberries from the three treatment groups (*p* > 0.05) (Table 1).

**Table 1:** Summary of mean values and standard deviation (SD) for the sensory evaluation on the strawberries cultivated from C (no PGPR); T1 (0.24% PGPR); and T2 (0.48% PGPR).

**Type of testing**	**Attributes**		**C**	**T1**	**T2**
Liking/Disliking 9-point Hedonic scale (1: Dislike extremely ~ 9: Like extremely)	Overall liking (N.S.)	Mean	6.92	6.92	6.92
		(SD)	(1.67)	(1.44)	(1.64)
	Appearance liking (N.S.)	Mean	6.75	6.90	6.79
		(SD)	(1.94)	(1.59)	(1.74)
	Flavor liking (N.S.)	Mean	6.81	6.98	7.02
		(SD)	(1.72)	(1.58)	(1.58)
	Texture liking (N.S.)	Mean	6.40	6.75	6.73
		(SD)	(2.00)	(1.64)	(1.5)
Just about right (JAR) (1: Much too little ~ 5: Too much)	Sweetness (N.S.)	Mean	2.67	2.65	2.73
		(SD)	(0.68)	(0.68)	(0.72)
	Tartness (N.S.)	Mean	2.92	2.94	2.83
		(SD)	(0.59)	(0.5)	(0.51)
	Firmness (N.S.)	Mean	2.42	2.56	2.52
		(SD)	(0.72)	(0.61)	(0.58)

*N.S* not significant

## Discussion

The metabolic processes of PGPR, such as phytohormone production and nitrogen fixation, may promote plant growth directly or indirectly (Glick 1995; Su et al. 2017). Direct plant growth promotion by PGPR requires either giving the plant a bacterium-produced substance, such as phytohormones that behave as chemical messengers or accelerate the uptake of essential nutrients, like nitrogen, from the environment (Glick 1995; Su et al. 2017). However, PGPR indirectly promote plant growth by reducing or preventing negative impacts of one or more phytopathogenic organisms. This can be accomplished by generating antagonistic chemicals or inducing resistance against pathogens (Beneduzi et al. 2012; Glick 1995). The PGPR may use one or more of these processes to promote plant growth and development (Glick 1995).

In the present study, three PGPR species (*B. subtilis*, *B. amyloliquefaciens,* and *P. monteilii*) were evaluated for their effects on the soil microbiome of strawberry plants and any subsequent impact on fruit quality. The application of three PGPR promoted the growth of other microbes such as nitrogen-fixation as a part of a synergistic effect. This finding is consistent with the previous reports that PGPR can enhance the growth and function of other microorganisms (Dos Santos et al. 2020). Moreover, this study discovered the potential role of PGPR as a ripening agent of strawberries.

The natural environment of soil is considered one of the most diverse habitats comprising numerous bacteria, fungi, and larger organisms such as nematodes, insects, or rodents (Jacoby et al. 2017; Sergaki et al. 2018). Zhou et al., (2002) suggested that the high microbial diversity of soil environment makes it challenging to alter or achieve dominance of introduced microbes. Both the Chao 1 and Shannon indices indicated that there was no significant difference in microbial diversity between C, T1, and T2 groups (*p* > 0.05). The beta diversity also showed highly similarity (R: 0.01) among groups (Fig. 7). The results of the present study that showed no difference microbial diversity among groups align with previous findings by Zhou et al. (2002) that is difficult to alter dominant microbes. The Venn diagrams representing shared and unique taxa identified during the 10 month study period supports the challenge that the PGPR had colonized the soil microbiota. From the beginning of the study period (August 2020) and the end (May 2021) the numbers of unique taxa did not decrease or increase for the T1 and T2 groups. However, instead of observing the overall microbial diversity, other approaches such as identifying specific microbe were conducted to explore the prevalence and level of the PGPR, which were comparing the relative abundance of two PGPR genera, *Bacillus* and *Pseudomonas,* in the three groups.

The three PGPR used in this study are suggested to promote the growth of other microorganisms as part of a synergetic effect to enhance plant growth via nitrogen fixation (Glick 1995; Pirlak and Köse 2009; Jacoby et al. 2017; Hashem et al. 2019; Dos Santos et al. 2020). There were six nitrogen-fixing taxa found in all groups: *Neorhizobium*, *Paenibacillus*, *Roseiarcus*, *Rhodanobacter*, *Devosia*, and *Microvirga* (Rivas et al. 2002; Khan and Doty 2009; Beneduzi et al. 2010; Mousavi et al. 2014; Zilli et al. 2015; Dobrovolskaya et al. 2020) (Fig. 4). The role of the nitrogen-fixing bacteria is to convert organic nitrogen or nitrogen gas in the atmosphere, to a plant usable form, such as inorganic ammonia (ammonification). In the soil, the nitrogen-fixing bacteria around the host plant roots provided a symbiotic effect on both the plant and the beneficial bacteria. The fixed nitrogen is subsequently transported to other areas of the plant, where it is used to create plant tissues and supports plant development (Peoples et al. 1995).

The microbial population shift of beneficial soil bacteria promotes the plant growth by enhancing resistance to pathogenic bacteria, degrading complex organic compounds, and producing substances that enhance root production. The beneficial soil bacteria identified in the T1 and T2 groups throughout the study period were observed to evaluate the potential functions of the PGPR on promoting the growth of other beneficial soil bacteria (Fig. 5). The *Ramlibacter* and *Flavobacterium* genera exhibited a gradual growth from the beginning to the end of the study period in both treatment groups (T1 and T2).

*Ramlibacter *genus is considered a group of beneficial soil bacteria that degrades sulfamethoxazole (SMX) which is a common antibiotic that is difficult to dissipate and enters the farm environment through water or landfills. It becomes an environmental contaminant that potentially increases the rate of antibiotic-resistant infections (Rauseo et al. 2019; Zhang et al. 2021). The other functions of *Ramlibacter* are enhancing plant resistance to pathogenic bacteria (De Luca et al. 2011). *Flavobacterium* is associated with the ability to degrade complex organic compounds, known as biodegradation, around the rhizosphere to provide the plant with usable nutrients. The decomposed organic compounds are used for plant growth, as well as bio-degradation which is an essential property to reduce the activity of toxic chemicals (Kolton et al. 2016). Similarly, *Sandaracinaceae* which showed a gradual growth in the T1 group are considered PGPR according to reports (Mohr et al. 2012). This bacterial family is known to degrade complex organic materials, such as starch, chitin, and cellulose (Mohr et al. 2012; Sharma et al. 2016). *Variovorax* increased throughout the study period and provides plant nutrients by degrading polyhydroxyalkanoates (PHAs) which are emerging plastic substitutes considered to be ideal for food packaging applications due to their biodegradability, nontoxicity, thermoplastic, and impermeability to gases and moisture. *Variovorax* is reported to be a PHAs degrading agent through its abiotic and biotic hydrolysis. The extracellular hydrolase enzyme in the cell wall of *Variovorax* converts the polymer of PHAs into naturally degraded forms, such as water-soluble monomers and oligomers, which can be used as plant nutrients (Masood 2017). *Rhodanobacter* is a nitrogen-fixing bacterium that had a gradual population growth in the T1 group. *Rhodanobacter* produces indoleacetic acid (IAA), which enhances root production of the host plant, thereby increasing nitrogen uptake from the soil. (Khan and Doty 2009).

Biofertilizers are organic substances containing microbes that can colonize the rhizosphere, enhance plant nutrient uptake, and promote the availability of nutrients to plant root hairs (Dasgupta et al. 2021). Since organic produce farming tends to avoid or limit the application of synthetic fertilizers, growth regulators, and livestock feed additives, biofertilizers are considered alternatives with expected similar outcomes (Samtani et al. 2019). The major findings of this study suggest that the consortium of three PGPR species: *B. subtilis*, *B. amyloliquefaciens,* and *P. monteilii,* have potential roles as a biofertilizer by enhancing the growth of other microbes as part of a synergetic effect.

Pomological effects of the PGPR on the strawberry quality were assessed in this study. The most notable outcomes were the level of TSS and the strawberry color among three treatment groups (Fig. 8). The color of the strawberry was darker from the treatment group treated in the order of PGPR concentration (T2 > T1 > C). Moreover, the strawberries from the PGPR treated plants contained a higher level of TSS compared to the C and T1 groups. Since the level of sweetness is an indicator of distinct quality, it is suggested that the PGPR may contribute to a quality-enhancing aid (Fan et al. 2021). Fruit ripening is a series of physiological, molecular, and biochemical processes leading to alterations in fruit in color, TSS, TA, flavor, texture, and aroma. The higher values of the TSS and TA, and the darker color suggest more mature strawberries (Kour et al. 2018; Maduwanthi and Marapana 2019; Valero and Serrano 2013). Considering that the length of the cultivation period was identical for the strawberries from all the treatment groups, it can be suggested that the PGPR presumptively behaved as a ripening enhancer. Faster ripening can be a potential advantage that may enable farmers to release the product at the desired ripening stage and avoid heavy competition with other producers in markets (Maduwanthi and Marapana 2019). Although more trials and deeper analysis are required, the current finding of PGPR’s potential role on maturation of strawberries can be the foundation of further research.

The other quality assessments measuring the amount of volatile compounds related to fruity/sweet flavor were conducted using a GC/MS. Methyl butanoate (fruity/sweet) and ethyl 3-methylbutanoate (sweet) were significantly higher in the strawberries in the order of T2, T1, and C group (*p* < 0.05) (Fig. 9). Additionally, butyl acetate (fruity/sweet) was significantly higher in both the T2 and C groups. The three volatile compounds found significantly higher in the T2 group (methyl butanoate and butyl acetate) were also found in a study conducted by Zhang et al., (2009). This study focused on analyzing volatile compounds in different strawberry cultivars and suggested that the three volatile compounds developed sweet and fruity flavors in strawberries (Zhang et al. 2009). According to another study, the butanoic and acetic acids exhibited a positive correlation with the PGPR treated strawberry (Todeschini et al. 2018).

The results of sensory evaluation showed no significant differences among the three treatment groups. The analytical TSS measurement indicated a significant difference between the three groups whereas, the sensory evaluation did not show the difference. The gap between the sensory evaluation and the instrumental measurements was previously discussed by Nishinari et al. (2019). In the sensory evaluation of food, the search for a connection between sensory and instrumental evaluation has been a trend to obtain more complete information about a product (Meiselman 1994; Swiader and Marczewska 2021).

In this study, the group of three PGPR (*B. subtilis*, *B. amyloliquefaciens,* and *P. monteilii*) was evaluated for their potential roles as a biofertilizer, using multidisciplinary approaches including microbiome, pomological, volatile compounds, and sensory analyses. In addition, this study was conducted on a commercial large scale strawberry farm to assess the practical applications of PGPR that can be applied to other crops. Although the significant findings in this study support the potential benefits of PGPR, there is still little use of PGPR in agriculture, due to their short shelf-life in the formulation, and limited advertising and training available for farmers to apply the formulations of PGPR (Backer et al. 2018). Promotion of these products are needed to evaluate the functionality of potential PGPR found in this study to verify the outcomes of the present study. Future investigation such as metabolite analysis produced by rhizosphere supplemented with PGPR will be conducted to delineate the metabolomics effects of PGPR on strawberry plants.

## Supplementary Information

**Additional file 1: Table S1.** Nucleotide sequences of primer pairs used in this study. **Table S2. **The thermocycler conditions for PCR assay used in this study.

## Data Availability

The 16S rRNA sequences are available at the BioProject of the National Center for Biotechnology Information (NCBI); PRJNA783001.
