# Supplementary material for: Multidisciplinary evaluation of plant growth promoting rhizobacteria on soil microbiome and strawberry quality
Source: AMB Express. 2023 Feb 16;13:18. doi: 10.1186/s13568-023-01524-z (PMC9935790; doi:10.1186/s13568-023-01524-z)
Supplement: Supplementary file 1 — Additional file 1: Table S1. Nucleotide sequences of primer pairs used in this study. Table S2. The thermocycler conditions for PCR assay used in this study. [file 13568_2023_1524_MOESM1_ESM.pdf]

**AMB Express**

# Multidisciplinary evaluation of plant growth promoting rhizobacteria on soil microbiome and strawberry quality

Jun Haeng Nam, Alyssa Thibodeau, Yanping L. Qian, Michael C. Qian, Si Hong Park*

Department of Food Science and Technology, Oregon State University, 3051 SW Campus Way, Corvallis, OR 97331, USA

***Corresponding author**

Si Hong Park, Ph.D., Assistant Professor, Department of Food Science and Technology, Oregon State University, 3051 SW Campus Way, Corvallis, OR 97331; Tel: 541-737-1684; Fax: 541-737-1877; E-mail: sihong.park@oregonstate.edu

**Table S1.** Nucleotide sequences of primer pairs used in this study

| Species | Primer | Sequence | Amplicon size (bp) | Target gene | Reference |
|---|---|---|---|---|---|
| *B. subtilis* | ytcP-f | GCTTACGGGTTATCCCGC | 480 | *ytcP* | Kwon et al. 2009 |
| | ytcP-r | CCGACCCCATTTCAGACATATC | | | |
| *B. amyloliquefaciens* | amyE-f | ACAAGTTAGTCACATGGGTG | 800 | *amyE* | Wu et al. 2014 |
| | amyE-r | TGCGGAAGATAACCATTCAAAC | | | |
| *P. monteilii* | glnK-f | GTCACAGCCATCATCAAGCC | 150 | *glnK* | In this study |
| | glnK-r | CGACCACATATTCAGCACCG | | | |

**Table S2.** The thermocycler conditions for PCR assay used in this study

| | ***B. subtilis*** | | ***B. amyloliquefaciens*** | | ***P. monteilii*** | |
|---|---|---|---|---|---|---|
| | **Temperature** | **Time** | **Temperature** | **Time** | **Temperature** | **Time** |
| Pre-denaturation | 94 ℃ | 5 min | 94 ℃ | 5 min | 94 ℃ | 5 min |
| Denaturation | 94 ℃ | 30 s | 94 ℃ | 30 s | 94 ℃ | 1 min |
| Annealing | 50 ℃ | 1 min | 50 ℃ | 1 min | 53 ℃ | 30 s |
| Extension | 72 ℃ | 30 s | 72 ℃ | 1 min | 72 ℃ | 30 s |
| Number of cycles | 35 | | 30 | | 40 | |
| Final extension | 72 ℃ | 5 min | 72 ℃ | 1 min | 72 ℃ | 5 min |
